# Supplementary material for: Taqman PACMAN: a simple molecular approach for positive rapid antigen test confirmation during periods of low prevalence
Source: Microbiol Spectr. 2024 Apr 3;12(5):e04073-23. doi: 10.1128/spectrum.04073-23 (PMC11064490; doi:10.1128/spectrum.04073-23)
Supplement: Fig. S1 — NAAT testing results in Nova Scotia spanning timelines relevant for this study. [file spectrum.04073-23-s0001.pdf]

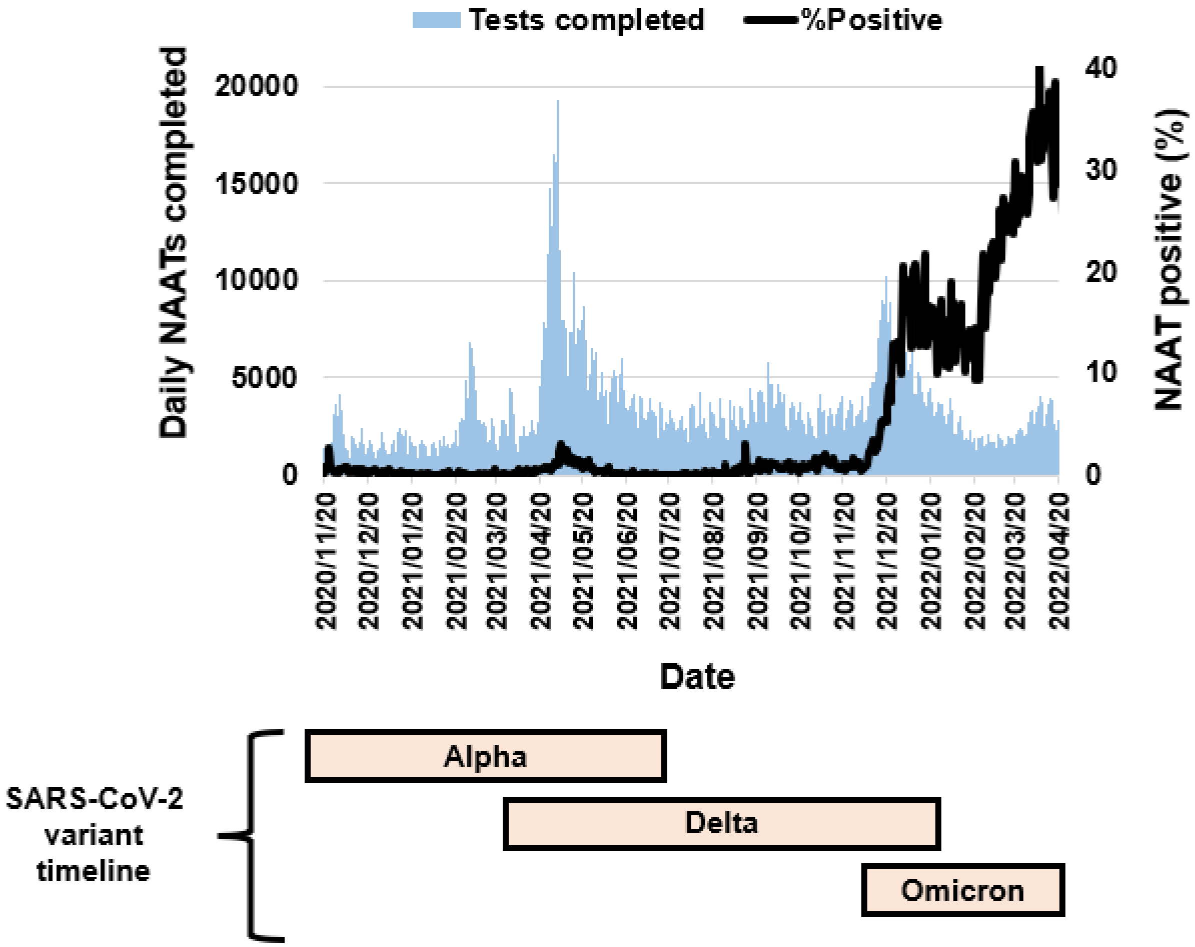

Figure S1. NAAT testing results in Nova Scotia spanning timelines relevant for this study. Timelines for circulating predominant SARS-CoV-2 variants of concern are depicted by orange boxes below the graph.
