# Supplementary material for: Taqman PACMAN: a simple molecular approach for positive rapid antigen test confirmation during periods of low prevalence
Source: Microbiol Spectr. 2024 Apr 3;12(5):e04073-23. doi: 10.1128/spectrum.04073-23 (PMC11064490; doi:10.1128/spectrum.04073-23)
Supplement: Fig. S2 — Correlation between threshold cycle values obtained with real-time RT-PCR with and without nucleic acid extraction. [file spectrum.04073-23-s0002.pdf]

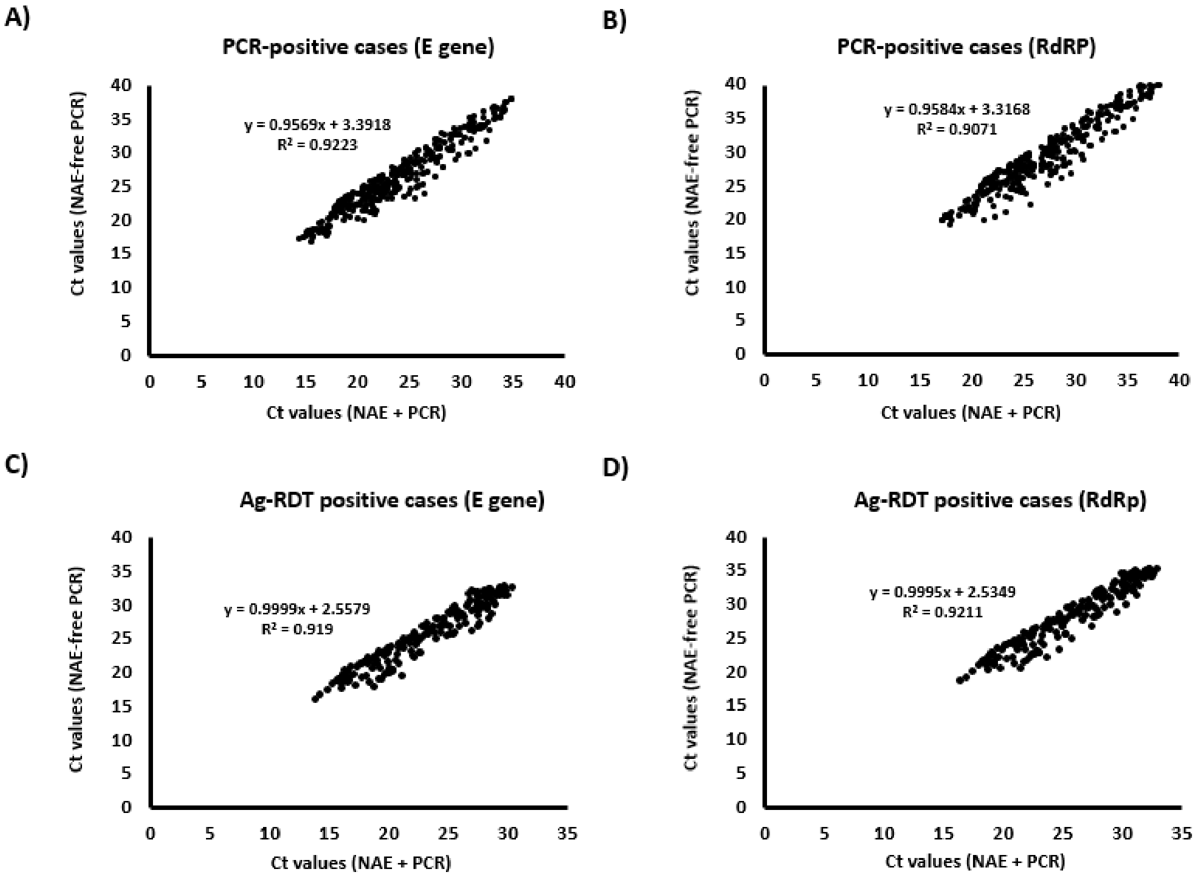

Figure S2. Correlation between threshold cycle (Ct) values obtained with real-time RT-PCR with and without nucleic acid extraction (NAE). A) E gene and B) RdRp Ct values obtained by comparing RT-PCR in the presence or absence of NAE using 338 concordant positive specimens from NAAT-positive individuals. C) E gene and D) RdRp Ct values obtained by comparing RT-PCR with or without NAE obtained from 257 positive NAAT specimens recollected from individuals that tested positive with Ag-RDTs.
