# Supplementary material for: Taqman PACMAN: a simple molecular approach for positive rapid antigen test confirmation during periods of low prevalence
Source: Microbiol Spectr. 2024 Apr 3;12(5):e04073-23. doi: 10.1128/spectrum.04073-23 (PMC11064490; doi:10.1128/spectrum.04073-23)
Supplement: Table S1 — Summary of threshold cycle values from the 14 discrepant results. [file spectrum.04073-23-s0003.pdf]

Table S1. Summary of threshold cycle (Ct) values) from the 14 discrepant results obtained when comparing RT-PCR with and without nucleic acid extraction (NAE) using commercial NAAT-positive specimens.

Specimen	NAE+		NAE-	
	E	RdRp	E	RdRp
1	35.27	38.37	ND	ND
2	35.47	38.57	ND	ND
3	35.75	38.85	ND	ND
4	35.75	38.85	ND	ND
5	36.16	39.26	ND	ND
6	36.27	39.37	ND	ND
7	36.66	38.76	ND	ND
8	36.89	39.80	ND	ND
9	36.10	39.20	ND	ND
10	36.14	37.24	ND	ND
11	36.61	39.71	ND	ND
12	36.63	39.73	ND	ND
13	36.91	39.79	ND	ND
14	36.95	39.05	ND	ND

ND = not detected
